# Rapid continuous microwave-assisted synthesis of silver nanoparticles to achieve very high productivity and full yield: from mechanistic study to optimal fabrication strategy

**DOI:** 10.1007/s11051-014-2843-y

**Published:** 2015-01-13

**Authors:** Grzegorz Dzido, Piotr Markowski, Anna Małachowska-Jutsz, Krystian Prusik, Andrzej B. Jarzębski

**Affiliations:** 1 Department of Chemical Engineering and Process Design, Faculty of Chemistry, Silesian University of Technology, Ks. M. Strzody 7, 44-100 Gliwice, Poland; 2 Department of Inorganic, Analytical Chemistry and Electrochemistry, Faculty of Chemistry, Silesian University of Technology, Ks. M. Strzody 7, 44-100 Gliwice, Poland; 3 Department of Environmental Biotechnology, Faculty of Energy and Environmental Engineering, Silesian University of Technology, Ks. M. Strzody 5, 44-100 Gliwice, Poland; 4 Institute of Materials Science, University of Silesia, Bankowa 12, 40-007 Katowice, Poland; 5 Institute of Chemical Engineering, Polish Academy of Sciences, Bałtycka 5, 44-100 Gliwice, Poland

**Keywords:** Silver nanoparticles, Microwave, Polyol process

## Abstract

Systematic studies of silver nanoparticle synthesis in a continuous-flow single-mode microwave reactor using polyol process were performed, revealing that the synthesis is exceptionally effective to give very small metal particles at full reaction yield and very high productivity. Inlet concentration of silver nitrate or silver acetate, applied as metal precursors, varied between 10 and 50 mM, and flow rates ranged from 0.635 to 2.5 dm^3^/h, to give 3–24 s reaction time. Owing to its much higher reactivity, silver acetate was shown to be far superior substrate for the synthesis of small (10–20 nm) spherical silver nanoparticles within a few seconds. Its restricted solubility in ethylene glycol, applied as the solvent and reducing agent, appeared to be vital for effective separation of the stage of particle growth from its nucleation to enable rapid synthesis of small particles in a highly loaded system. This was not possible to obtain using silver nitrate. All the observations could perfectly be explained by a classical LaMer–Dinegar model of NPs’ formation, but taking into account also nonisothermal character of the continuous-flow process and acetate dissolution in the reaction system. The performed studies indicate an optimal strategy for the high-yield fabrication of metal particles using polyol method.

## Introduction

In the last two decades, metal and oxide nanoparticles (NPs) have attracted much attention owing to their unique size- and shape-dependent physical, chemical, catalytic, and biological properties and extensive application prospects in modern industries and medicine (Dzido et al. 2008; Manoth et al. 2009; Rai et al. 2009; Kundu et al. 2009a, b); Khajeh and Sanchooli 2011; Sharma et al. 2011; Wang et al. 2012; Breitwieser et al. 2013; Kundu and Jayachandran 2013; Bryaskova et al. 2014; Wu et al. 2014). For this reason, the effective processes of the large-scale production of size-controlled NPs are of major importance (Kundu et al. 2009c; Aksomaityte et al. 2013; Yang et al. 2013).

Among the various physical and chemical methods applied to synthesize metal NPs, the polyol-mediated synthesis, first proposed by the University of Paris group, offers a number of important advantages: it is convenient, environmentally friendly, and versatile (Fivet and Brayner 2013). In essence, it makes use of the reduction of inorganic precursors in liquid polyols, i.e., polyhydroxy alcohols such as ethylene glycol, polyvinyl alcohol, and even glycerol, or their water solutions, which can dissolve to some extent ionic inorganic compounds. However, the rate of chemical reduction with polyols appeared to be low compared with aqueous systems (Zhao et al. 2010). Therefore, it had to be enhanced to become practically feasible, and it was achieved by a microwave (MW) irradiation of reaction systems proposed by Komarneni et al. (1995), and later adopted by the others (Pastoriza-Santos and Liz-Marzan 2002; Gao et al. 2005; Tsuji et al. 2005; Li and Komarneni 2006; Jiang et al. 2006; Kundu et al. 2008a). In effect, the reaction time could be reduced to minutes or even seconds (Kundu et al. 2008b; Horikoshi et al. 2010; Raghunandan et al. 2011). However, in addition to the increase in reaction rate, the use of MW resulted in more uniform heating of the whole reaction system. This enabled us achieve better control of the NPs’ nucleation and growth, directly affecting the particle size distribution (PSD).

The most significant limitation of the MW-assisted techniques, seen from the scale-up and hence NPs’ fabrication perspective, is that the penetration depth of microwaves is constrained typically to a few centimeters (Opembe et al. 2013). It renders the scale up of batch reactors hardly possible, but it is large enough for a rational design of continuous-flow synthesis of metal NPs to achieve high yields (Tu and Liu 2000; Groisman and Gedanken 2008; Gjuraj et al. 2012). Moreover, if a tubular reactor is installed in a single-mode microwave cavity, then a homogenous irradiation, and hence uniform heating, can be obtained (Nishioka et al. 2011; Horikoshi et al. 2013). The steady and uniform irradiation, while somewhat overlooked until most recently, appears to be vital to achieve a reproducible control of the continuous fabrication of NPs (Nishioka et al. 2011).

While the scores of previous studies focused on establishing effective procedures for the synthesis of specific NPs in batch-type systems, we are familiar with only a few reports on the continuous reaction systems, contributing to the development of synthetic methodologies of metal NPs adaptable to industrial needs (Gold et al. 2007; Horikoshi et al. 2010; Nishioka et al. 2013). Therefore, we deemed it important to carry out more systematic studies of the continuous-flow metal NPs’ synthesis using an originally designed reactor with uniform MW irradiation. In this paper, we report the results of silver nanoparticle synthesis to propose a methodology rather than a direct fabrication procedure, based on understanding the mechanisms of their formation, and not just shear experimental findings. We studied the effects of main process and reaction system variables: precursor type and its concentration, temperature, residence time, and also density of MWs’ power input, on the formation of AgNPs to elucidate the mechanisms of their rapid synthesis. In effect, we identified and clarified some of the observed characteristics of AgNPs using the classical LaMer–Dinegar (LMD) (LaMer and Dinegar 1950) mechanism of NPs’ formation, but seen from a nonisothermal, continuous-flow perspective. Owing to considerable interest in AgNPs as antibacterial agents, we also examined antibacterial properties of the AgNPs-based suspensions, directly obtained from the reported continuous process.

## Experimental

### Microwave reactor system

**Fig. 1:**
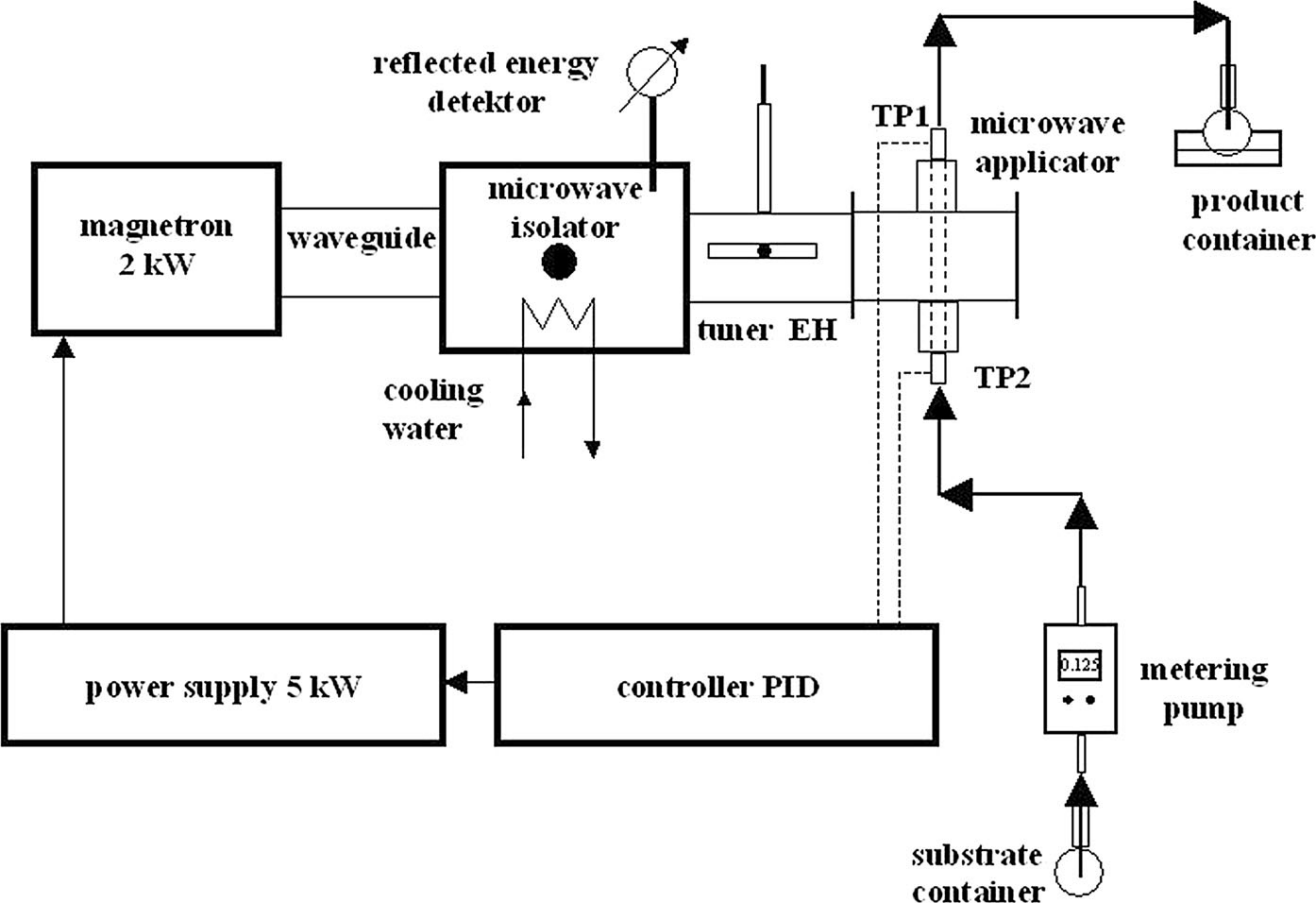
Experimental set-up of the microwave apparatus applied in synthesis of silver nanoparticles

The continuous synthesis of AgNP was realized in a set-up schematically shown in Fig. 1 using a commercial 2 kW single-mode microwave system (ERTEC, Poland) operating at 2.45 GHz frequency. Microwaves were transferred from the magnetron, through a microwave isolator and EH tuner, to the microwave applicator, i.e., a cavity with a PTFE tubular reactor (8 mm i.d., 42 mm length of irradiated section) mounted inside. The reactants mixture was fed continuously using a fine metering pump, at controlled flow rates of 0.318, 0.635, 1.27, and 2.5 dm^3^/h, to achieve the preset values of residence time (3, 6, 12 and 24 s) in the irradiation zone. The power was supplied continuously, and not intermittently as had been applied previously. Its value was controlled to achieve the preset outlet temperatures (in the range of 90–170 ± 1 °C depending on Ag precursor) and was measured by a thermocouple mounted at the reagents’ outlet. The diameter of tubing outside the reactor was reduced to 2-mm i.d. to speed up the reactants transport to a well-cooled collection cup.

Inside the reactor the temperature rose continuously from room temperature along the tube, owing to the continuous MW irradiation, and the available power allowed to obtain heating rates even of over 40 °C/s, hardly attainable in conventional reactors. However, that rate seemed to be essential for a high-yield production of metal nanoparticles in a cost effective process.

### Continuous synthesis of silver nanoparticles

The reaction solution was prepared in two steps, first a specified amount of polyvinylpyrolidone (PVP, K-25 or K-30 type, Mw of 24 and 40 kDa, respectively, extra pure, Roth), determined to give molar ratio Ag: PVP (monomeric unit) 1:7, was dissolved under stirring at 80 °C, primarily in ethylene glycol (EG, 99.9, Aldrich) However, in separate experiments also in 1,2-propanediol (reagent grade, POCH, Poland) or 1,4-butanediol (+99 %, Acros), to obtain a clear solution which was cooled down to room temperature. Then, a specified amount of a Ag metal precursor, either silver acetate (AgOAc, +99 %, Alfa Aesar) or silver nitrate (AgNO_3_, +99.9 % POCH) was dissolved. In the case of AgNO_3_, a clear liquid was obtained in the concentration range of 10-50 mM, whereas for AgOAc, a clear solution was observed only for 10 mM content. Therefore, for its larger (20 and 50 mM) concentrations, it was additionally sonicated shortly to avoid overheating, and then continuously magnetically homogenized at room temperature before delivery into the reactor with flow rates as given above. After rapid cooling of the suspension, the samples were taken and analyzed for silver ions content using differential pulse voltammetry (DPV) method (vide infra) to determine its conversion, and thus the value of Ag^0^ yield. Each experiment was repeated thrice. The samples obtained were held at room temperature and stability of selected ones was analyzed after one moth storage. The suspensions were also assayed for the antibacterial activity.

### Instruments and product analyses

Size of nanoparticles was determined by a dynamic light scattering (DLS) technique using a Zetasizer S90 instrument (Malvern Instrument, UK). UV–Vis absorption spectra were recorded using a Hitachi U-2800A spectrophotometer with quartz cuvette. HRTEM morphological analysis of silver particles was performed using a JEOL JEM 3010 field emission microscope working at an accelerating voltage of 300 kV equipped with energy dispersive X-ray spectroscope (EDX). First, a sample of silver nanoparticles suspension was diluted in acetone and then centrifuged at 30 k/min. Supernatant liquid was removed, and after diluting the remains with water, a drop of the colloidal suspension was placed on a holey carbon film covering copper grid and allowed to evaporate at room temperature. The crystalline phases of the particles were examined by X-ray diffractometry (XRD) using an X-Pert Pro apparatus (Phillips, Netherlands) and Cu Kα radiation (*λ* = 0.15418 nm).

**Fig. 2:**
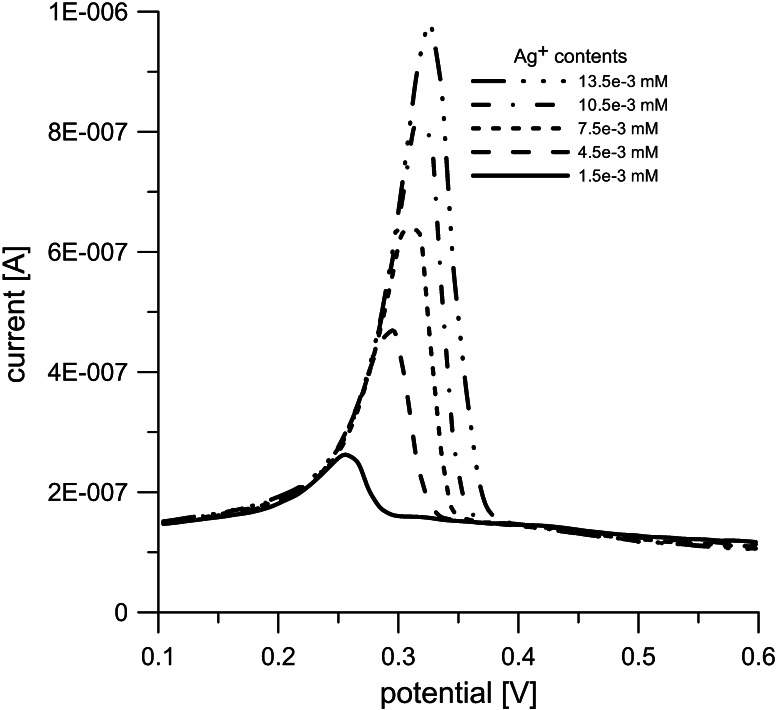
Voltamograms of liquids with specified concentrations of silver ions

DPV method applied to determine silver ions content in the suspension on the reactor’s exit made use of the selective ion oxidation at the working electrode (glassy carbon), with silver/silver chloride (Ag/AgCl) and platinum electrode being a reference and an auxiliary electrode, respectively. A mixture of Britton-Robinson buffer (pH 4.10) with ethylene glycol (99/1, v/v) was used as a supporting electrolyte. Figure 2 shows the voltamograms obtained from the liquids with a specified concentration of silver ions. The areas under the curves are proportional to the concentration of Ag^+^ ions. In this way, a calibration curve could be developed, to be later applied to determine Ag^+^, and hence Ag^0^ content, to give the reaction yield. The accuracy of analytic determinations ranged from 0.4 to 3.6 %.

Antimicrobial activity of AgNPs was evaluated using disk diffusion test (Kirby-Bauer method) for two different kinds of bacteria: gram-positive *Bacillus subtilis* and gram-negative *Escherichia coli*. Pure bacteria strains were isolated from environmental samples. Then the isolated strains were suspended in a sterile physiological salt solution to obtain the bacterial suspensions which were used to prepare bacterial cultures with a spread plate method. Prior to the analyses, silver agents were diluted with a sterile physiological salt solution to obtain their 100 and 25 % concentrations. Sterile blotting paper disks moistened with the prepared silver agents’ dilutions, were placed in the middle of agar plates with inoculated bacteria. *B. subtilis* culture was incubated for 48 h at 20 °C, while *Escherichia coli* culture was incubated for 24 h at 37 °C. After the incubation period, zones of inhibition were measured. Five test repetitions were carried out for both bacteria strains, and the arithmetic means of the measured results were calculated (data not shown).

## Result and discussion

### General observations

**Fig. 3:**
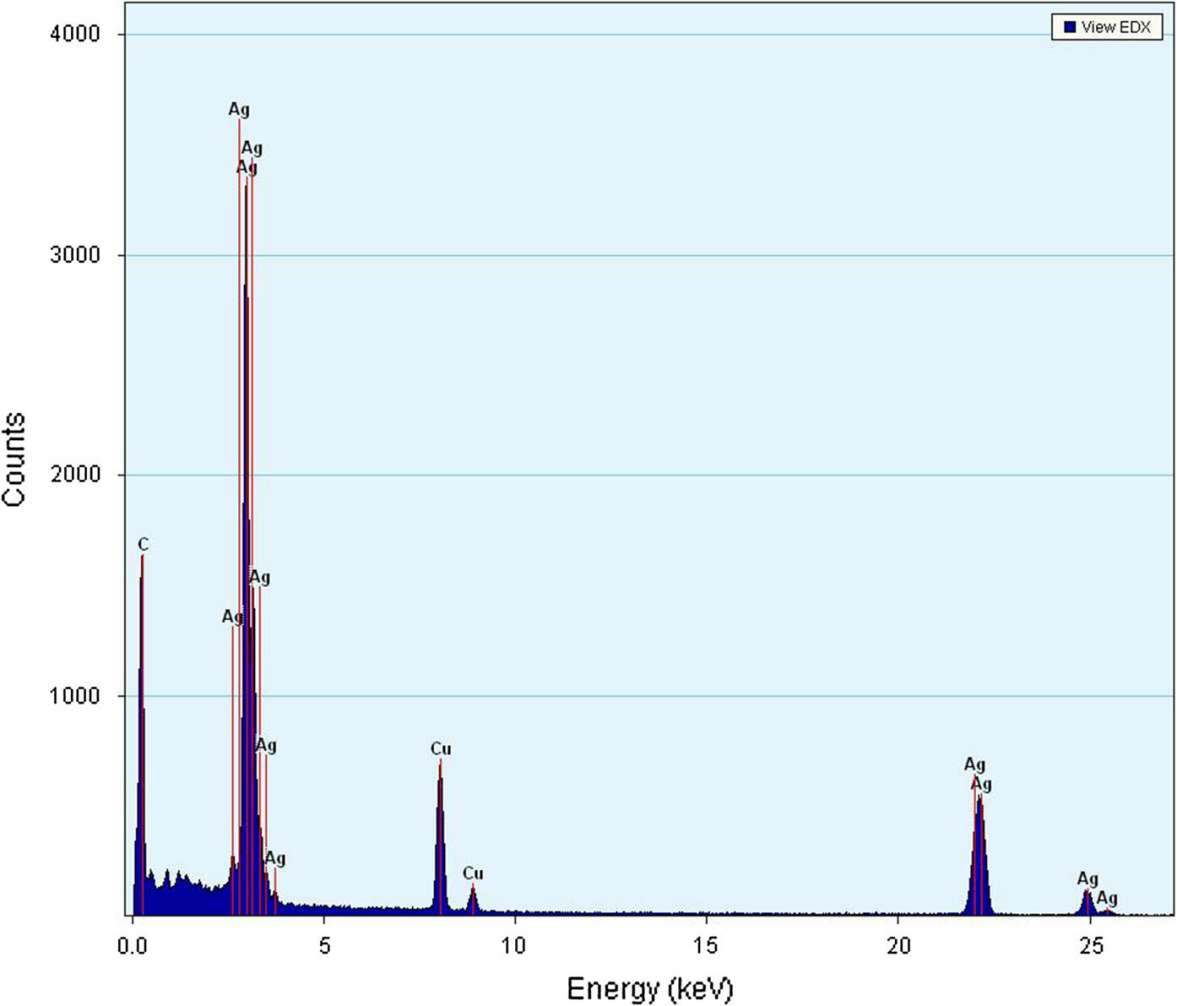
EDX analysis for the silver NPs (T9)

Preliminary experiments showed that for the continuous-flow synthesis of silver NPs in 3–24 s under continuous MW heating (thus nonisothermal conditions), the exit temperatures had to be held in the ranges of 150–180 °C and 90–175 °C, for silver nitrate and silver acetate, respectively. This allowed the region of the colloidal system formation to be kept within a microwave cavity, as could be checked from the silver ions’ content as determined by DPV measurements. In this way, we could conveniently control the process. EDX analysis of the colloidal mixture confirmed the abundant presence of silver particles, as deduced from the spectrum displayed in Fig. 3, where the C and Cu peaks come from the carbon-coated copper TEM grid. In addition, UV-absorption spectra of the colloidal systems, examined for the presence of surface plasmon resonance (SPR) bands of AgNPs at ~419 nm, corroborated silver NPs’ formation (Creighton and Eadon 1991; Mulvaney 1996). Selected samples were also examined using TEM and XRD. The obtained patterns appeared to exhibit pronounced peaks, which were found to correspond with crystallographic planes of face-centered cubic silver NPs. These patterns were also used to evaluate the size of crystalline particles from Scherrer equation. DLS analysis of the selected colloidal systems (T9, T14) after 1-month storage confirmed their quite good stability. Moreover, using the same procedure as applied for the preparation of TEM samples, we obtained solid samples, which could easily be redispersed in the liquid.

Table 1 lists the conditions of silver nanoparticles’ production and results of the experiments performed (average of three measurements). Already, a superficial inspection of the values of AgNPs yields (determined from DPV data) indicates that Ag-nitrate is much less reactive then Ag-acetate, and hence longer residence times and/or higher temperatures were necessary to obtain high yields of particles. Thus, at least 12 s were necessary to obtain ca. 95 % conversion of silver nitrate into metal particles for the exit temperature of 170 °C (*cf.* T9), whereas for silver acetate, full reaction yield was achieved in less than six seconds at the exit temperature of 150 °C (*cf.* T13, T17, and T19 in Table 1). Also, interestingly enough, for larger silver nitrate concentrations, under otherwise identical conditions, larger conversion of Ag^+^ was measured (*cf.* T2 and T9), a clear indicative of some sort of autocatalytic effect, more recently suggested by the Watzky et al. (2008) model of nanocluster formation, and not predicted by the LMD mechanism of NPs’ formation. This effect was not seen for silver acetate, most likely due to its restricted solubility in EG, and dissolution directly affecting the overall kinetics of NPs’ formation.

**Table 1:** Synthesis conditions for the preparation of silver nanoparticles

Sample	Stabiliser precursor	Outlet temperature (°C)	Mean residence time (s)	z-average diameter (nm)	SD^a^ (nm)	PDI^b^	Initial precursor conc. (mM)	Substrate conversion (%)
T1	PVP K30 AgNO_3_	149	12	160	2.9	0.2	10	56
T2		171	12	88.0	3.3	0.5	10	63
T3	PVP K30 AgOAc	90	12	78.9	1.9	0.3	10	42
T4		150	6	37.5	1.5	0.5	10	>99
T5		155	12	49.4	3.5	0.3	10	95
T6		155	24	50.9	1.7	0.3	10	>99
T7		169	24	54.2	1.3	0.3	10	>99
T8	PVP K30 AgNO_3_	171	6	126	5.2	0.2	50	78
T9		171	12	138	6.1	0.2	50	94
T10		174	24	146	3.3	0.2	50	94
T11	PVP K30 AgOAc	150	6	23.6	0.8	0.6	20	>99
T12		90	12	63.9	0.5	0.5	50	45
T13		150	6	40.0	1.7	0.5	50	>99
T14		151	12	41.7	0.5	0.5	50	>99
T15		150	24	42.6	1.4	0.5	50	>99
T16		170	24	55.6	2.5	0.4	50	>99
T17	PVP K25 AgOAc	150	3	34.2	1.1	0.5	10	>99
T18		150	6	28.8	0.6	0.6	10	>99
T19		152	3	30.5	1.1	0.6	20	>99
T20		149	6	30.9	1.2	0.6	20	>99
T21		150	12	30.4	0.3	0.6	20	>99
T22		150	12	31.0	2.4	0.6	20	>99
T23		90	12	75.3	2.6	0.6	20	53
T24		90	24	51.4	1.8	0.5	20	66
T25		150	24	31.7	0.7	0.5	20	>99
T26	PVP K25 AgOAc	150	6	35.3	0.9	0.6	20	99^c^
T27	PVP K25 AgOAc	150	6	20.3	2.5	0.6	20	85^d^

^a^Standard deviation

^b^Polydyspersity index

^c^1,2-Propanediol

^d^1,4-Butanediol

### Impact of synthesis and process parameters

**Fig. 4:**
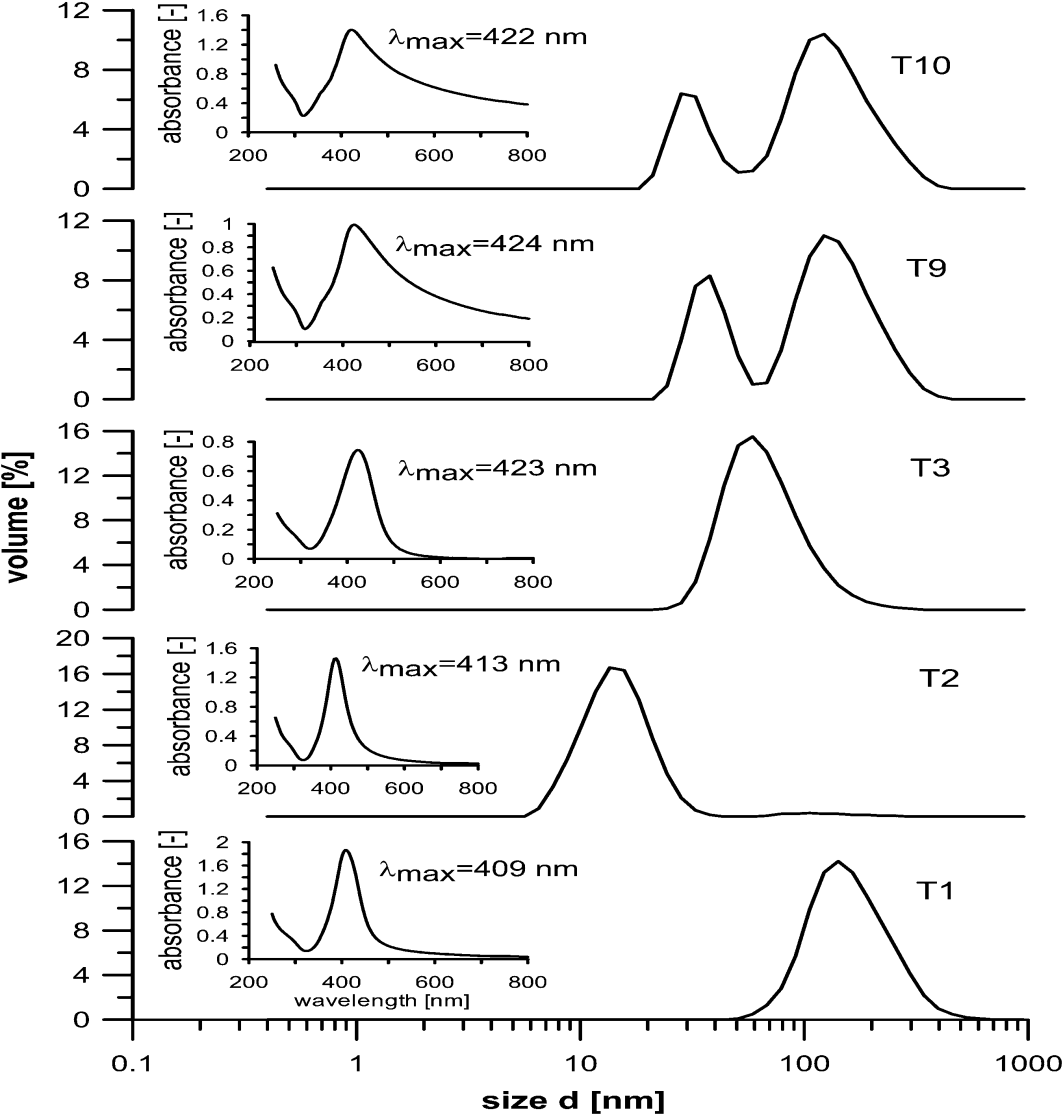
Effect of ‘*thermal history*’ on size distribution histograms (by DLS) and UV–Vis spectra of silver particles synthesized using Ag-nitrate and Ag-acetate

Experiments showed that the exit temperature of ~170 °C was a reasonable compromise for the synthesis AgNPs using Ag-nitrate, and it was ~150 °C for Ag-acetate. In perfect agreement with LMD model, the mean sizes of particles (hydrodynamic diameter detected by DLS) produced at lower exit temperatures of 149 and 90 °C, respectively for Ag-nitrate and Ag-acetate, were large, ca. 140 and 65 nm (*cf.* T1 and T3 in Table 1, T1 and T3 in Fig. 4), and the reactions were far from completion, despite a fairly long residence time (12 s). When the rate of substrate reduction was increased, by fixing the exit temperature at 171 °C, the size of particles obtained from Ag-nitrate decreased to 15–20 nm (T2 in Fig. 4), which could be ascribed to the increase in the rate of reduction, and hence of nucleation, resulting in a larger number of original nuclei, but the reaction yield increased only from 56 to 63 % (*cf.* Table 1). All these were observed for Ag-nitrate concentration of 10 mM. However, for its large (inlet) content (50 mM), which is of most practical interest, we saw a development of two-size distributions, both of them fairly narrow, with maxima at about 35 and 110 nm (T9 in Fig. 4), and the Ag^0^ yield achieved in 12 s was ca. 94 %. Similar bimodal size distribution patterns of AgNPs were also observed before in a low-temperature continuous-flow process (Dzido and Jarzębski 2011), and the formation of smaller particles was ascribed to the oscillation-related particles’ abrasion, more intense at higher concentrations (Zhu et al. 2004).

**Fig. 5:**
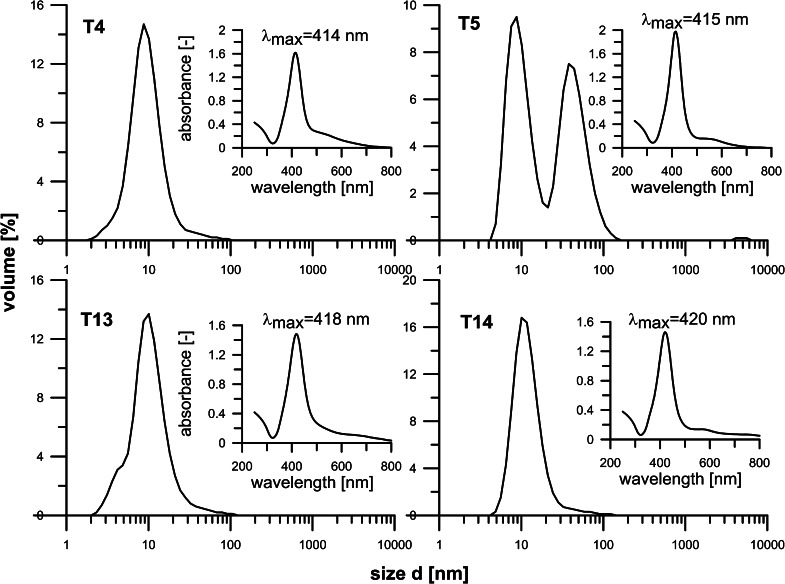
Particle size distributions histograms (by DLS) and UV–Vis spectra of samples produced with 10 mM (T4, T5) and 50 mM (T13, T14) Ag-acetate inlet content

However, a more plausible explanation, perfectly in accordance with LMD model, which we propose herein, is that these bimodal size distributions of NPs directly stem from a nonisothermal nature of the realized continuous-flow process, and hence, the presence of two distinct regimes of particle formation prevailing in central and exit sections of the reactor. In the region of low and medium temperatures, the rate of substrate reduction is low and hence the concentration of species generated by the reaction of reduction and hydrolysis cannot easily reach a supersaturation level, needed for nucleation (Fivet and Brayner 2013). This facilitates their attachment to few particles already present in the system, ending up in the synthesis of large particles, as predicted by LMD model. The second regime, which prevails in a high-temperature (center and/or outlet) region, features very fast rate of substrate reduction and rapid increase in the concentration of final species to the value exceeding a critical supersaturation, i.e., nucleation level. In effect, it triggers very fast nucleation, which immediately lowers concentration below that of nucleation. If the concentration of final species remains higher than the saturation level, then a slow growth of particles follows. This mechanisms results in the formation of small particles, also seen in the PSD patterns (Fig. 4), in agreement with the LMD model predictions (Blosi et al. 2011; Fivet and Brayner 2013). Clearly, this picture is obscured by both temperature- and MW-stimulated particle oscillations which facilitate coalescence and aggregation of smaller particles into larger ones. However, on the whole, this portrayal is also supported by a larger amount of bigger NPs obtained at longer reaction time, under otherwise identical conditions (*cf.* T9 and T10 in Fig. 4). As already pointed out, Ag-acetate appeared to be much more reactive than Ag-nitrate, and hence very small particles, of less than 10 nm, could be obtained in just 3 s at full reaction yield, and even at the exit temperature of 150 °C. However, closer analyses of the PSD (hydrodynamic diameter by DLS) patterns showed that again the trends observed are in good agreement with the predictions of LMD model. At low inlet concentrations (10 mM) and under very vigorous microwave heating, to achieve the exit temperature of 150 °C in just 3 s, particles with size distribution ranging from 5 to 10 nm were synthesized at full reaction yield and with a minute content of those <5 nm (T17, data not shown), a clear sign of very rapid nucleation. If a MW power supply was reduced by half (T18, 6 s), still narrower PSD pattern was achieved, with a majority of particle sizes being in the range of 3–4 nm (by DLS, data not shown). Clearly, too powerful irradiation (short reaction time) appears to be detrimental for the formation of uniform particles. We ascribe it to both the MW-induced oscillations and collisions, ending up in the aggregation of very fine particles (of 2–3 nm) to give those 5–8 nm in size, However, also to vigorous, repeated multiple-step dissolution and crystallization phenomena. They disrupt a dynamic equilibrium between reduction, nucleation, and growth, deemed to be critical for the synthesis of uniform particles (Blosi et al. 2011). All these experiments were performed using K25 PVP as a capping agent. When K30 PVP was applied, PSDs appeared to be somewhat wider (*cf.* T4 Fig. 5), which points to the importance of effective capping in the rapid synthesis of NPs. Worth noting is that application of PVP with even shorter chain (Mw 8 kDa) had no appreciable effect on particle sizes. Surprisingly enough, at lower power input, and hence longer reaction times (T5, 12 s and T6, 24 s), particles with bi- (T5, Fig. 5) and even tri-size (T7) distributions in the range of 8–80 nm were obtained (not shown), when PVP with a longer chain (K30), and hence less capping ability, was applied. We believe that the mechanism of particle formations is exactly the same as that discussed above for Ag-nitrate system, but owing to much higher reactivity of silver acetate–EG system, the particles were considerably smaller. In this respect, it is noteworthy that low temperature (90 °C, T3) synthesis in the same time resulted in the formation of particles with a narrow PSD, similar to that corresponding to larger particles seen in the bimodal distributions of T5 and T6, in good agreement with the observations made for T9 and T10 (Fig. 4).

**Fig. 6:**
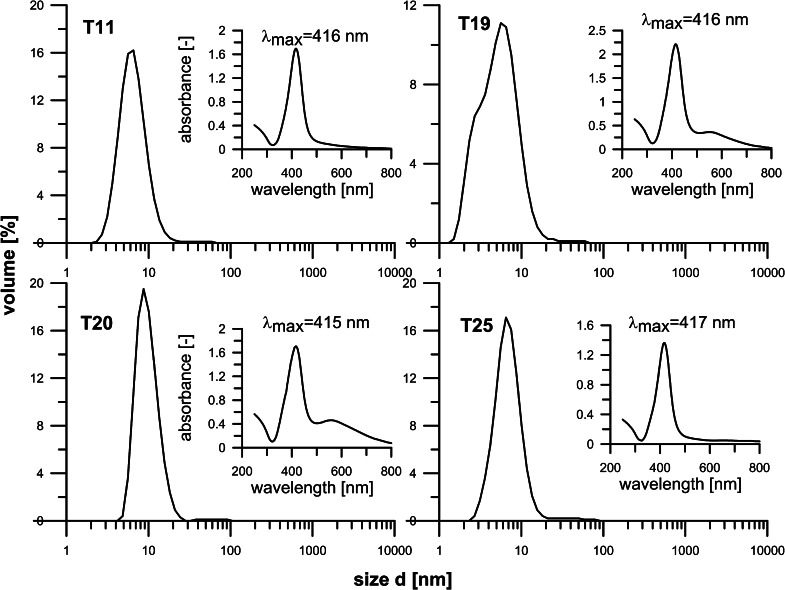
Particles size distributions histograms (by DLS) and UV–Vis spectra of samples produced using Ag-acetate (20 mM) and different reaction times

In order to increase productivity, experiments were also carried out for larger inlet concentrations (20 and 50 mM) of Ag-acetate, i.e., well above its solubility limit in EG, which to the best of our knowledge was not explored before toward the synthesis of silver particles. On the whole, all these experiments ended up in the synthesis of very small particles, typically of less than 10 nm in size and at full reaction yield, which took us by surprise, but can be of major practical significance. Moreover, while the size distribution patterns (by DLS) of particles obtained from most rapid process (3 s, 150 °C exit temp.) appeared to be somewhat spread out (T19, Fig. 6) those of particles formed more slowly (6 or 24 s) tended to be more narrowly distributed and NPs were quite small, less or about 10 nm in size (T11, T20, and T25, Fig. 6), which again is quite attractive. However, more importantly, the same observations were also made for the systems with Ag-acetate content of 50 mM. Again, silver particles achieved from a rapid process (6 s, T13) appeared to be very small, yet their PSD was not so narrow (Fig. 5), in contrast to those obtained from less intensive heating (12–24 s), which were more narrowly distributed and quite small, prevalently in the range of 8–20 nm (*cf.* T14, Fig. 5). The results obtained again seem to comply with the LMD mechanism of particle formation, provided that the limited solubility of Ag-acetate in EG, and hence a need for its dissolution in the reaction system, is taken into account in addition to high reactivity. The rate of the former is not significantly affected by the change in temperature, but by vigorous particle oscillations. In effect, in a medium and high temperature region, where the rate of metal substrate reduction could potentially be very high, the rate of dissolution controls the rate of substrate reduction and hydrolysis and thus concentration of the final species, which cannot easily reach a critical supersaturation level. Yet owing to a continuous dissolution, and hence supply of Ag-acetate into the reaction zone, the concentration of these species can be maintained higher than a saturation level in a large section of the reactor. On the whole, this results in the attachment of more slowly generated species to very small particles formed in large amount in initial stages, resulting in their slow growth further downstream. In effect, the steps of nucleation and growth are effectively separated, in agreement with the postulates of LMD model for the synthesis of uniform particles (LaMer and Dinegar 1950; Fivet and Brayner 2013). However, not less importantly perhaps, the identified mechanism of silver nanoparticles’ formation from Ag-acetate also paves the way to a robust, cost-effective fabrication method of very high yield and productivity.

Preliminary experiments showed that 1,2-propanediol and 1,4-butanediol can also be applied as solvents and reducing agents to give silver particles of less than 10 nm in size in just 6 s using Ag-acetate of 20 mM. Numerous particles obtained using 1,4-butanediol appeared to be exceptionally small (2–4 nm by DLS, not shown) but the reaction yield was lower (85 %, *cf.* Table 1), which we ascribe to larger viscosity and hence lower diffusivity of species, which tends to control the process (Park et al. 2011).

### Characterization of nanoparticles and antibacterial properties of suspensions

Analyses of UV–Vis spectra of the synthesized particles showed that most of them exhibit a typical peak at around 419 nm associated with SPR adsorption by silver nanoparticles. More detailed inspection of the spectra revealed, however, that peak intensities depend quite notably on the applied substrate. They were narrow and clearly pronounced in all Ag-acetate-derived NPs, irrespective of its inlet concentration (Figs. 5, 6 insets) and residence/reaction time, whereas for Ag-nitrate-derived particles, this picture appeared to be more complex. They were narrow and pronounced for low (10 mM) inlet concentration but wide, with loci of maximum being red-shifted to 422–424 nm, when they were equal to 50 mM (*cf*. T1 and T2 against T9 and T10 in Fig. 4 insets). The latter spectra usually imply the presence of both small and larger particles, which, indeed were clearly seen in the corresponding PSD patterns (Fig. 4). A fourfold extension of reaction time (from 6 to 24 s) had little effect on SPR spectra, which indicates that the mechanisms of NPs’ formation, and thus PSD patterns did not change. For the Ag-acetate-derived particles, the same extension of reaction time (6–24 s) also resulted in minor changes in SPR peaks; they were even further narrower and red-shifted from 415 to 417 nm and from 418 to 420 nm, for 20 and 50 mM samples, respectively (T20, T25 in Fig. 6, and T13, T14 in Fig. 5). These observations correspond to changes in the PSD patterns (Figs. 5, 6), and corroborate those reported before, yet for much longer reaction times (Dzido and Jarzębski 2011; Kim et al. 2006).

**Fig. 7:**
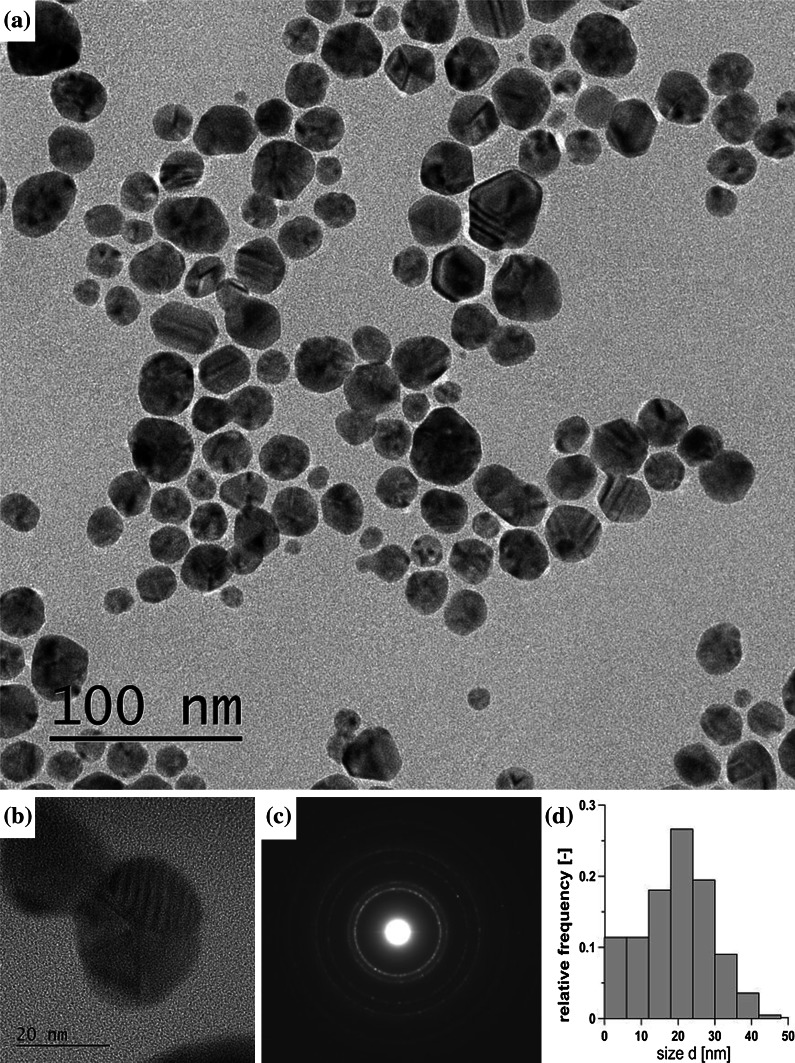
Characteristics of Ag NPs fabricated in continuous flow process (T14): **a** TEM image, **b** HRTEM enlargement, **c** corresponding SAED pattern, **d** PSD obtained by counting 420 particles

An analysis of TEM images corroborated the results of DLS predictions of particle sizes. The genuine (by TEM) size of the particles obtained proved to be slightly larger than those evaluated from the DLS method and displayed in Figs. 4, 5, and 6, but significantly smaller than predicted by z-average estimate (Table 1). It can be seen from images of T14 (Ag-acetate, 12 s, 50 mM) displayed in Fig. 7 that silver particles fabricated in the rapid continuous-flow process are small and fairly spherical, some of them twinned and polycrystalline, as could be inferred from SAED diffraction pattern.

**Fig. 8:**
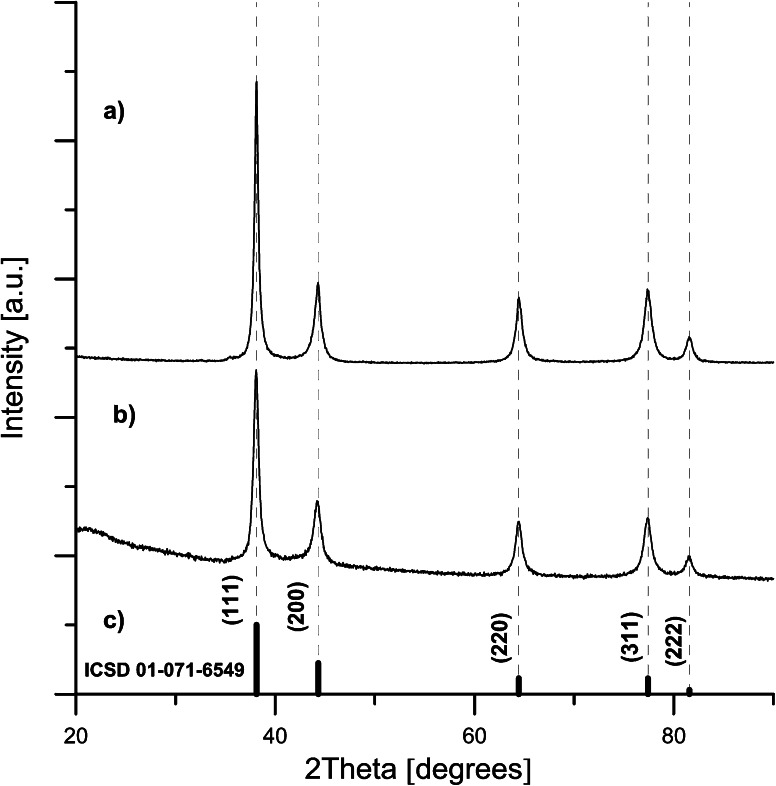
XRD patterns of Ag NPs: **a** sample T14, **b** sample T9, **c** silver reference (ICSD card 01-071-6549)

In this respect, worth noting are the pronounced diffraction peaks clearly seen in XRD patterns of T9 and T14 (*cf.* Fig. 8). The results indicate the crystalline structure of the Ag NPs with observed peaks closely matching those of pure silver (ICSD card 01-071-6549, reflections at 2*θ* = 38.12°, 44.31°, 64.45°, 77.41°, and 81.56°). They correspond, respectively, to the (1 1 1), (2 0 0), (2 2 0), (3 1 1), and (2 2 2) crystallographic planes of face-centered cubic Ag crystals. The size of crystalline particles calculated from Scherrer equation (*d* = *Kλ/β*cos(*θ*) where *d* is the average crystal grain size, *λ* is the wavelength of the X-rays, *β* is the broadening of reflection due to crystallite size effect, *θ* is the diffraction angle of a particular reflection*, K* = 0.9), was of an average diameter in the range of about 13–15 nm for both T9 and T14 sample. These estimates are in good agreement with the maxima of PSDs obtained from TEM and DLS analyses of T14 (*cf.* Figs. 5, 7).

**Fig. 9:**
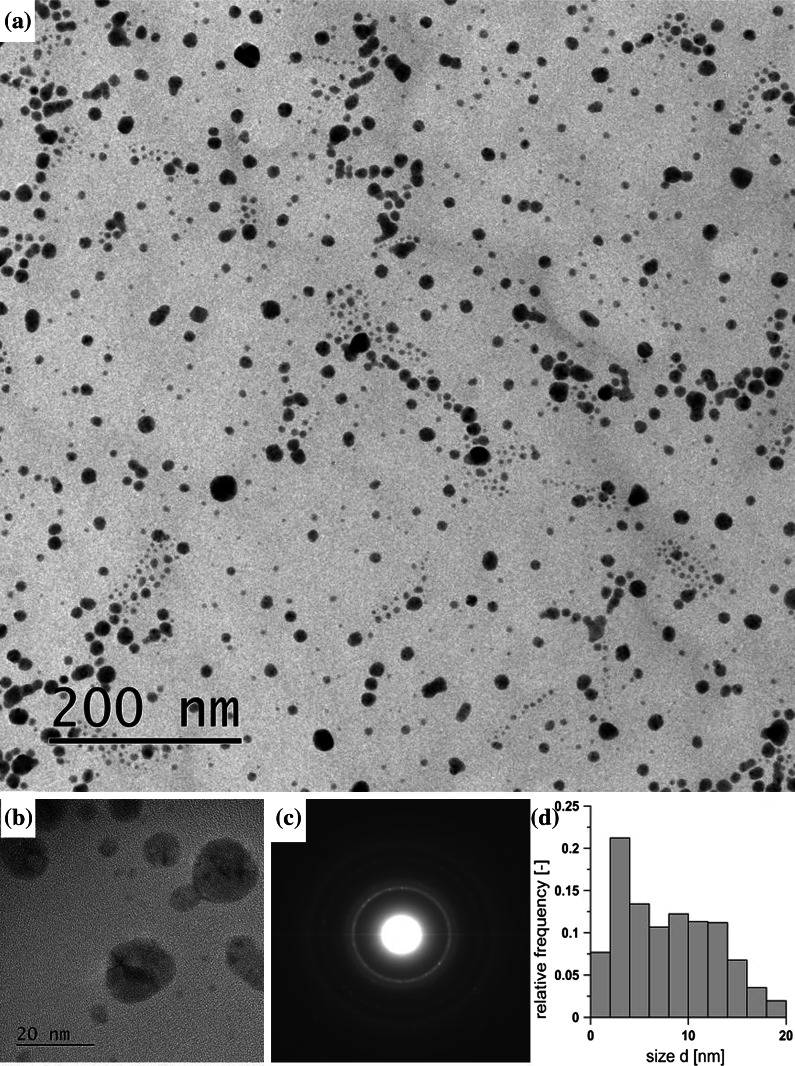
Characteristics of Ag NPs fabricated in continuous flow process (T27): **a** TEM image, **b** HRTEM enlargement, **c** corresponding SAED pattern, **d** PSD obtained by counting 780 particles

TEM image observations also confirmed that application of 1,4-butanediol results in the synthesis of very fine particles (T27, Fig. 9), notably smaller than those obtained using EG, in accord with DLS-based measurements, yet with significantly wider size distribution, also in agreement with the predictions of DLS method (Table 1).

**Fig. 10:**
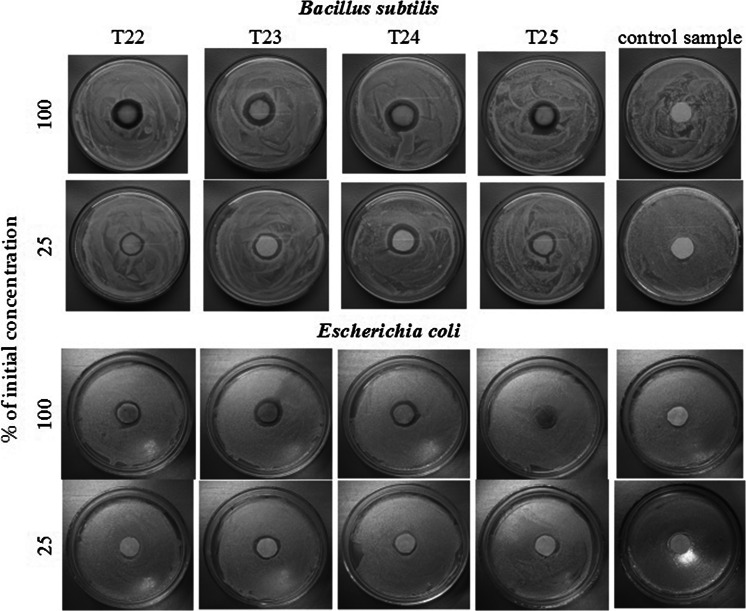
Bactericidal activity against gram-positive *Bacillus subtilis* and gram-negative *Escherichia coli* of selected silver nanoparticle suspensions: as obtained from fabrication (100 %) and after fourfold dilution (25 %)

Investigations of antibacterial activities of four nanoparticle suspensions (T22–T25) obtained directly from the process (20-mM Ag-acetate series) and also of their fourfold dilution solutions proved that they all exhibit bactericidal activity, somewhat stronger toward gram-positive than gram-negative bacteria. However, more important perhaps is that bactericidal activity proved stronger for T23- and T24-derived suspensions, i.e., those with large content of unreduced ions (Ag^0^ yield of 53 and 66 %). This could be inferred from larger sizes of inhibition zones of the respected samples and also their diluted solutions (Fig. 10).

The exact mechanism of silver nanoparticles antimicrobial activity is not completely understood. The Ag NPs can affect the generation of cell membrane and cell wall, inhibit protein synthesis, attach and interact with DNA or disturb metabolic processes (Dallas et al. 2011; Guzman et al. 2012). Most of the recent reports relate it to the AgNPs’ impact on cell membrane and its damage due to disturbance of major cell membrane functions, such as permeability, respiration, or mechanical protection (Gutarowska et al. 2012; Yoksan and Chirachanchai 2010). The bactericidal activity of AgNPs can be intensified by silver ions Ag^+^. Positively charged silver ions interact with negatively charged cell membrane (Rai et al. 2009; Yoksan and Chirachanchai 2010) causing its shrinkage and its detachment from the cell wall. Silver ions can also deactivate cell proteins and bind to DNA, thus inhibiting its replication (Sondi and Salopek-Sondi 2004). Cell metabolic functions can also be damaged due to reaction between silver ions and functional groups of enzymes containing sulfur, nitrate, or oxygen (Choi et al. 2008).

## Conclusions

Continuous-flow microwave-assisted polyol method of metal particles synthesis was shown to be exceptionally effective to give small, uniform spherical silver nanoparticles within seconds at very high yield and productivity. However, the size of nanoparticles appeared to be strongly dependent on the applied substrate, with silver acetate unexpectedly performing significantly better in the proposed process than silver nitrate. It was found to be caused by its much higher reactivity and also limited solubility in ethylene glycol, which contrary to popular belief, appeared to be crucial to afford very high productivity of NPs’ fabrication at full reaction yield. The limited solubility of silver acetate facilitates the separation of particle growth from its nucleation, and hence it is vital for the continuous production of uniform particles in a highly concentrated system. All this could be concluded from the mechanistic analysis of NPs’ formation performed using the classical LaMer–Dinegar model, but taking into account both nonisothermal nature of the proposed process and acetate dissolution in the reaction system. We believe that a mechanistic portrayal of the proposed process may also be applied to elucidate and boost other processes of metal NPs’ fabrication.
